# Evaluation of Factors Predictive of Efficacy Among Patients With Complicated Urinary Tract Infection and/or Acute Pyelonephritis

**DOI:** 10.1093/ofid/ofae375

**Published:** 2024-07-16

**Authors:** Sujata M Bhavnani, Jeffrey P Hammel, Christopher M Rubino, Angela K Talley, Paul B Eckburg, Kathryn Liolios, Vipul K Gupta, Paul G Ambrose, Kamal A Hamed, David A Melnick

**Affiliations:** Institute for Clinical Pharmacodynamics, Inc ., Schenectady, New York, USA; Institute for Clinical Pharmacodynamics, Inc ., Schenectady, New York, USA; Institute for Clinical Pharmacodynamics, Inc ., Schenectady, New York, USA; Spero Therapeutics, Inc., Cambridge, Massachusetts, USA; Spero Therapeutics, Inc., Cambridge, Massachusetts, USA; Institute for Clinical Pharmacodynamics, Inc ., Schenectady, New York, USA; Spero Therapeutics, Inc., Cambridge, Massachusetts, USA; Institute for Clinical Pharmacodynamics, Inc ., Schenectady, New York, USA; Spero Therapeutics, Inc., Cambridge, Massachusetts, USA; Spero Therapeutics, Inc., Cambridge, Massachusetts, USA

**Keywords:** complicated urinary tract infection, efficacy, ertapenem, pharmacokinetic-pharmacodynamic, tebipenem

## Abstract

**Background:**

Antibiotic treatment for complicated urinary tract infections (cUTI)/acute pyelonephritis (AP) is often followed by recurrent bacteriuria in the absence of clinical symptoms. To understand factors predictive of clinical and microbiologic outcomes in patients with cUTI/AP, multivariable analyses were undertaken using pooled data from a global, phase 3 cUTI study.

**Methods:**

Using data from 366 tebipenem pivoxil hydrobromide– and 378 ertapenem-treated patients from the Study to Assess the Efficacy, Safety and Pharmacokinetics of Orally Administered Tebipenem Pivoxil Hydrobromide (SPR994) Compared to Intravenous Ertapenem in Participants With Complicated Urinary Tract Infection (cUTI) or Acute Pyelonephritis (AP) infected with Enterobacterales uropathogens, multivariable analyses for dichotomous efficacy endpoints were performed using logistic regression and pharmacokinetic-pharmacodynamic relationships were evaluated.

**Results:**

Urinary tract anatomical disorders and functional urinary tract or metabolic disorders were predictive of nonresponse across all efficacy endpoints assessed at test-of-cure (TOC) and late follow-up (LFU) visits, with greater impact on overall and microbiologic than clinical nonresponse. Independent variables predictive of increased probabilities of successful overall response at TOC and microbiologic response at TOC or LFU were baseline creatinine clearance >50 mL/min and baseline pathogen fluoroquinolone susceptibility. Infection with a phenotypic extended-spectrum beta-lactamase–positive Enterobacterales pathogen was predictive of reduced probabilities of success for microbiologic response at LFU and clinical response at TOC. Meaningful relationships between efficacy endpoints and plasma pharmacokinetic-pharmacodynamic indices were not identified.

**Conclusions:**

Reductions of overall and microbiologic response in patients with cUTI/AP were associated with anatomical or functional urinary tract disorders, but not with the magnitude or duration of plasma antibiotic exposure. Results of these analyses serve to advance our understanding of factors predictive of outcome in patients with cUTI/AP.

## Teaser

Multivariable analyses were undertaken using Phase 3 study data from TBP-PI-HBr- and ertapenem-treated patients with cUTI/AP. Reductions of overall and microbiologic response were associated with anatomical or functional genitourinary disorders, but not with plasma drug exposure within a PK-PD-optimized range

After antibiotic treatment for complicated urinary tract infections (cUTI) or acute pyelonephritis (AP), patients often experience recurrent bacteriuria even when they do not exhibit clinical symptoms [1–14]. Determining factors that predict failure in clinical and microbiologic response among patients with cUTI/AP enrolled in clinical studies could inform clinical trial design for this indication.

In the phase 3 clinical trial, Study to Assess the Efficacy, Safety and Pharmacokinetics of Orally Administered Tebipenem Pivoxil Hydrobromide (SPR994) Compared to Intravenous Ertapenem in Participants With Complicated Urinary Tract Infection (cUTI) or Acute Pyelonephritis (AP) (ADAPT-PO) (NCT03788967), investigators compared the efficacy of oral tebipenem-pivoxil hydrobromide (TBP-PI-HBr) to intravenous (IV) ertapenem in hospitalized patients with cUTI/AP. This prospective, randomized, and double-blind clinical trial found that TBP-PI-HBr was noninferior to IV ertapenem in terms of clinical, microbiologic, and overall (composite of both clinical and microbiologic) responses at the end-of-treatment (EOT), test-of-cure (TOC), and late follow-up (LFU) assessments in the microbiologic intention-to-treat population [11]. To better understand which factors influenced the efficacy of treatment in these patients, we evaluated pooled data from TBP-PI-HBr– and ertapenem-treated patients with cUTI/AP in the ADAPT-PO study. The objectives for these analyses included identifying predictive factors of clinical and microbiologic efficacy endpoints using multivariable analyses, examining whether these factors varied between the treatment groups, and exploring the relationships between efficacy endpoints and pharmacokinetic-pharmacodynamic (PK-PD) indices for each drug.

## METHODS

### Analysis Populations and Efficacy Endpoints

Data for TBP-PI-HBr– and ertapenem-treated patients who were microbiologically evaluable (ME) and infected with Enterobacterales pathogen(s) at baseline (a subpopulation of the microbiologic intention-to-treat population from the ADAPT-PO study [11]) were considered for the analyses performed. Analyses were performed using ME populations at EOT (days 7–10, or up to day 14 for bacteremic patients), TOC (day 19 ± 2), or LFU (day 25 ± 2), which were referred to as ME-EOT, ME-TOC, and ME-LFU, respectively, herein. To be included in any one of these analysis populations, patients were required to have a baseline Enterobacterales pathogen, corresponding minimum inhibitory concentration (MIC) data, tebipenem pharmacokinetic data, or sufficient covariate data to estimate ertapenem PK, and to be evaluable for at least 1 efficacy endpoint. The analysis was limited to patients with Enterobacterales pathogen(s) identified at baseline because this pathogen group is the most common among patients with cUTI/AP. Dichotomous efficacy endpoints included clinical, microbiologic, and overall response endpoints at EOT and TOC, as well as clinical and microbiologic response at LFU. The protocol-defined criteria for inclusion in the ME populations and the previously-described efficacy endpoints have been described elsewhere [11].

### Multivariable Analyses for Efficacy

Multivariable analyses to evaluate factors predictive of efficacy were performed using R version 4.0.4 [15]. Multivariable logistic regression models for the previously-described dichotomous efficacy endpoints were constructed, with candidate independent variables including baseline patient demographics and disease characteristics based on data from the previously-described analysis populations. The infrequency of interactions between individual candidate independent variables and treatment group across the efficacy endpoints was assessed to determine the appropriateness of constructing multivariable models using data from both TBP-PI-HBr– and ertapenem-treated patients. Multivariable models were developed using the forward inclusion of independent variables with an entry criterion of largest improvement of Akaike's information criterion (AIC) [16], if any. Multivariable models, when developed, were limited to no more independent variables than 10% of the total number of failures for the given efficacy endpoint [11]. Wald *P-*values were used to describe the statistical significance of the independent variables retained in the final models.

Interactions between treatment group and candidate independent variables were assessed in the context of the multivariable models. Any such interactions that were significant at the 0.10 level were noted and taken into consideration for inclusion as additional model covariates for the assessment of PK-PD relationships for efficacy described below.

The multivariable models for efficacy endpoints were used to assess if PK-PD indices within treatment groups were associated with efficacy endpoints in the context of covariate adjustments for independent variables in the models and for any interacting variables subsequently identified. The PK-PD index evaluated for the TBP-PI-HBr–treated patients was the ratio of tebipenem free-drug plasma concentration–time curve from 0 to 24 hours on day 1 (AUC) to the MIC (AUC:MIC ratio), which was normalized to tau (τ) (AUC:MIC ratio × 1/τ). A tebipenem protein binding estimate of 42% (ie, a free fraction of 0.58) was used to generate day 1 free-drug plasma AUC values [17]. For this PK-PD index, τ represented the duration of the dosing interval (ie, 8 hours for a dosing regimen of every 8 hours) and the MIC was the tebipenem MIC for the baseline pathogen. The PK-PD index evaluated for the ertapenem-treated patients was percentage of time on day 1 that ertapenem free-drug plasma concentrations were above the MIC of the baseline pathogen (%T > MIC). Tebipenem free-drug plasma AUC:MIC ratio × 1/τ was determined using individual dosing histories, post hoc PK parameter estimates, and a previously-described population PK model for tebipenem [18].

Ertapenem free-drug plasma %T > MIC was determined using relevant patient characteristics and a published population PK model for ertapenem. The population PK model described by Lakota et al. was a linear 3-compartment model with total body weight as a covariate on clearance and body surface area as a covariate on central volume [19]. This model was refined and creatinine clearance (CLcr) normalized to body surface area (mL/min/1.73 m^2^) was added as a predictor of the interindividual variability in ertapenem clearance. Using the concentration-dependent relationship between ertapenem free-drug and total-drug plasma concentrations based on data from Majumdar et al. [20], the refined population PK model, and previously-described patient characteristics, free-drug plasma concentration-time profiles were generated for ertapenem-treated patients. From these concentration-time profiles over 24 hours and a baseline Enterobacterales MIC value, free-drug plasma %T > MIC was determined for each ertapenem-treated patient. A predictive performance exercise was conducted to determine whether the use of the population PK model, together with dosing history and demographic information alone, was appropriate for the generation of ertapenem PK parameters and free-drug plasma %T > MIC. The methods for this assessment, which was undertaken since PK data were not available for ertapenem-treated patients in the ADAPT-PO study, are described in the Supplementary material.

In addition to the previously-described PK-PD indices, baseline MIC was evaluated by treatment group, and tebipenem free-drug plasma AUC was also assessed for TBP-PI-HBr–treated patients, each in the multivariable context. For a given efficacy endpoint and treatment-specific PK-PD index or MIC, the covariate-adjusted assessment of their relationship was performed using the log-transformed continuous form of the PK-PD index or MIC as an independent variable added onto the model for the efficacy endpoint developed as described previously. An optimized 2-group form of the PK-PD index or MIC was assessed in a similar fashion, with the threshold of the 2-group variable determined using the first split of a univariable classification tree for each dichotomous efficacy endpoint. To assess the impact of the covariate adjustments, the univariable associations were also assessed for comparison.

Evaluation of 200 simulated versions of ertapenem free-drug plasma %T > MIC with respect to their associations with the efficacy endpoints were performed as sensitivity analyses, where each simulation represented a different random application of individual variability in model PK parameters (ie, using a different seed for each simulation).

## RESULTS

### Analysis Population

A total of 827 ME patients (415 TBP-PI-HBr–treated patients and 412 ertapenem-treated patients) with cUTI/AP from the ADAPT-PO study had a baseline pathogen with an MIC value, PK data, and were evaluable for at least 1 efficacy endpoint. Of these patients, 744 (366 in the TBP-PI-HBr arm and 378 in the ertapenem arm) had an Enterobacterales pathogen at baseline and were included in the pooled population (ie, the analysis population). As would be expected, the majority of these patients with cUTI had anatomical disorders of the urinary tract (53.1%), functional urinary tract or metabolic disorders (20.7%), or prior urinary tract instrumentation or surgery (13.0%). Summary statistics for categorical and continuous baseline patient demographics and disease characteristics by study arm and in this pooled population are shown in Supplementary Tables 1 and 2, respectively. Listings of terms used to define urinary tract anatomical disorders, functional urinary tract or metabolic disorders, and urinary tract instrumentation/procedures are summarized in Supplementary Table 3.

Summaries of observed successful responses for efficacy endpoints are provided in Table 1. The percentage of patients with cure for clinical response at EOT, TOC, or LFU (ie, sustained clinical cure for the latter) ranged from 91.6 to 99.4% and 93.5 to 98.9% for TBP-PI-HBr– and ertapenem-treated patients, respectively. The percentage of patients with favorable microbiologic response and overall response at EOT was 99.7 and 99.2%, respectively, for TBP-PI-HBr–treated patients and 100 and 98.9%, respectively, for ertapenem-treated patients. The percentage of patients with favorable microbiologic response at TOC or LFU or overall response at TOC ranged from 65.6 to 67.9% for TBP-PI-HBr–treated patients and 68.6 to 75.7% for ertapenem-treated patients.

**Table 1.** Summary of Successful Responses for Efficacy Endpoints by Visit Based on Data From Patients in the ME Analysis Population Infected With Enterobacterales Pathogen(s) at Baseline

Efficacy Endpoint	Percentage of Successful Responses (n/N)		
	TBP-PI-HBr–Treated Patients	Ertapenem-Treated Patients	Pooled Treatments
Clinical response at EOT	99.4 (361/363)	98.9 (365/369)	99.2 (726/732)
Clinical response at TOC	93.8 (319/340)	97.1 (335/345)	95.5 (654/685)
Clinical response at LFU	91.6 (293/320)	93.5 (301/322)	92.5 (594/642)
Microbiologic response at EOT	99.7 (362/363)	100 (369/369)	99.9 (731/732)
Microbiologic response at TOC	67.9 (231/340)	75.7 (261/345)	71.8 (492/685)
Microbiologic response at LFU	65.6 (210/320)	68.6 (221/322)	67.1 (431/642)
Overall response at EOT	99.2 (360/363)	98.9 (365/369)	99.0 (725/732)
Overall response at TOC	66.8 (227/340)	74.2 (256/345)	70.5 (483/685)

Abbreviations: EOT, end-of-treatment; LFU, late follow-up; ME, microbiologically evaluable; TBP-PI-HBr, tebipenem-pivoxil hydrobromide; TOC, test-of-cure.

Summary statistics for tebipenem day 1 free-drug plasma AUC, baseline MIC, and free-drug plasma AUC:MIC ratio × 1/τ and ertapenem baseline MIC and day 1 free-drug plasma %T > MIC based on data from patients in the ME analysis population infected with Enterobacterales pathogen(s) at baseline are shown in Table 2. A high percentage of TBP-PI-HBr– and ertapenem-treated patients achieved nonclinical PK-PD targets associated with net bacterial stasis and a 1-log_10_ CFU reduction from baseline [21–25].

**Table 2.** Summary Statistics for Tebipenem Day 1 Free-Drug Plasma AUC, Baseline MIC, and Free-Drug Plasma AUC:MIC Ratio × 1/τ or Ertapenem Baseline MIC and Day 1 Free-Drug Plasma %T > MIC Based on Data From the Patients in the ME Analysis Population^a^ Infected With Enterobacterales Pathogen(s) at Baseline

Summary Statistic	Tebipenem (n = 366)			Ertapenem (n = 378)	
	Day 1 Free-Drug Plasma AUC (mg•h/L)^b^	Baseline MIC (µg/mL)	Day 1 Free-Drug Plasma AUC:MIC Ratio x 1/τ^c^	Baseline MIC (µg/mL)	Day 1 Free-Drug Plasma %T > MIC^d,e^
Mean (%CV)	43.5 (70.8)		321 (96.2)		95.1 (17.3)
Median or MIC_50_, MIC_90_ (Min, Max)	35.9 (6.64, 388)	0.015, 0.12 (≤0.004, 2)	238 (3.31, 2223)	0.008, 0.12 (≤0.002, >8)	99.6 (0, 99.6)

Abbreviations: AUC, area under the curve; CV, coefficient of variation; EOT, end-of-treatment; LFU, late follow-up; ME, microbiologically evaluable; MIC, minimum inhibitory concentration; PK-PD, pharmacokinetic-pharmacodynamic; TOC, test-of-cure.

^a^The ME population contains all patients in any one of the populations, including ME-EOT, ME-TOC, or ME-LFU.

^b^Tebipenem free-drug plasma AUC was calculated using a protein binding estimate of 42%.

^c^The percentage of TBP-PI-HBr–treated patients that achieved median tebipenem free-drug plasma AUC:MIC ratio × 1/τ targets associated with net bacterial stasis of 6.42 and a 1-log_10_ CFU reduction from baseline of 9.56 for Enterobacterales isolates studied in a 1-compartment in vitro infection model [21] was 98.6 and 98.4%, respectively. The percentage of patients achieving median tebipenem free-drug plasma AUC:MIC ratio × 1/τ targets associated with the same endpoints (21.4 and 45.1, respectively) based on data for Enterobacterales isolates from a neutropenic acute pyelonephritis model [22] was 95.1 and 89.9%, respectively.

^d^Ertapenem free-drug plasma %T > MIC was determined using the concentration-dependent relationship between ertapenem free-drug and total-drug plasma concentrations based on data from Majumdar et al. [20].

^e^Studies with carbapenems carried out using data from a neutropenic murine thigh infection model were used to estimate the magnitude of the PK-PD index required for ertapenem endpoints. The percentage of ertapenem-treated patients that achieved ertapenem free-drug plasma %T > MIC targets associated with net bacterial stasis of 30% and a 1-log_10_ CFU reduction from baseline of 40% based on data for Gram-negative bacilli studied in a neutropenic murine thigh infection model [23–25] was 97.4 and 96.0%, respectively.

The results of the validation exercise provided in the Supplementary Materials demonstrated that the use of the typical values of the population PK parameters for ertapenem based on relevant patient characteristics have the ability to provide unbiased and reasonably precise estimates of free-drug plasma %T > MIC among ertapenem-treated patients in the analysis population.

### Interaction Assessments to Support Evaluation of Pooled Data

Several statistically significant univariable associations were identified between efficacy endpoints and the candidate independent variables for multivariable models. However, statistically significant interactions with treatment group were infrequent at the 0.10 level, suggesting that multivariable analyses based on the pooled dataset were ideal for constructing models based on these candidate independent variables (ie, relative to constructing separate models by treatment group). Assessments of univariable associations between efficacy endpoints and candidate independent variables for the multivariable analyses are shown in Supplementary Table 4. Results are presented for all efficacy endpoints except those assessed at EOT, for which there were insufficient numbers of failures to support univariable and multivariable analyses.

### Summary of Multivariable Analyses

A summary of independent variables and interactions appearing in final multivariable models for the efficacy endpoints is provided in Table 3. The most commonly appearing independent variables across the multivariable models for the efficacy endpoints assessed were baseline urinary tract anatomical disorders and/or functional urinary tract or metabolic disorders, which were predictive of reduced probabilities of successful response. Additional predictive patient factors that were assessed at baseline, including sex, body mass index, CLcr > 50 mL/min, diagnosis, bacteremia, baseline pathogen fluoroquinolone susceptibility, modified systemic inflammatory response syndrome (SIRS) criteria, and prior systemic antibiotics, appeared in some models and not others.

**Table 3.** Summary of Baseline Independent Variables Retained in Multivariable Models for the Efficacy Endpoints^a^

Baseline Independent Variable	Clinical Response		Microbiologic Response		Overall Response
	TOC	LFU	TOC	LFU	TOC
Increased age					
Sex (male)					
Increased body mass index				X^b^	X^b^
CLcr > 50 mL/min^c^			X^d^	X^d^	X^d^
Ethnicity					
Race					
Region					
Diagnosis of cUTI (potentially including AP) versus AP alone					
Positive extended-spectrum beta-lactamase resistance phenotype	X^b^			X^b^	
Fluoroquinolone susceptible phenotype			X^d^		X^d^
Trimethoprim-sulfamethoxazole susceptible phenotype		I^e^			
Bacteremia			X^d^		X^d^
Urinary tract anatomical disorders	X^b^	X^b^	X^b^	X^b^	X^b^
Functional urinary tract or metabolic disorders	X^b^		X^b^	X^b^	X^b^
Instrumentation at baseline	I^e^	I^e^			
Modified systemic inflammatory response syndrome criteria		X^d^			
Prior systemic antibiotics		X^b^			

Abbreviations: AP, acute pyelonephritis; CLcr, creatinine clearance; cUTI, complicated urinary tract infection; LFU, late follow-up assessed on day 25 ± 2; TOC, test-of-cure assessed on day 19 ± 2.

^a^Multivariable analyses for dichotomous endpoints were performed using logistic regression.

^b^Associated with a reduced probability of a successful response for the dichotomous efficacy endpoint.

^c^CLcr was calculated by Cockcroft Gault equation using serum creatinine data collected at baseline from the local laboratory [26].

^d^Associated with an increased probability of a successful response for the dichotomous efficacy endpoint.

^e^“I” indicated that an interaction with treatment group, when added to the AIC-selected model, was significant at the 0.10 level. Ethnicity, race, and region each had a very high majority of patients within a single category, and were unable to be studied for interactions with treatment group in the context of the multivariable models.

The final multivariable model for overall response at TOC is shown in Table 4, whereas models for the remaining efficacy endpoints are shown in Supplementary Table 5 to Supplementary Table 8. Baseline urinary tract anatomical disorders and/or functional urinary tract or metabolic disorders were commonly the most impressive factors with respect to magnitude and statistical significance in the models. Assessed among all patients in the analysis population, the mean reductions in model-predicted successful response percentages based on the combination of both factors versus neither factor (with other model variables remaining as observed for each patient) were 7.0 and 7.8% for clinical response at TOC and LFU, respectively, 29.2 and 31.2% for microbiologic response at TOC and LFU, respectively, and 27.9% for overall response at TOC. Therefore, the impact of urinary tract anatomical disorders and functional urinary tract or metabolic disorders was substantially greater for overall and microbiologic than clinical response. In some models, seemingly counterintuitive independent variables associated with improved response included bacteremia and/or the modified SIRS criteria at baseline. However, as Supplementary Table 9 demonstrates, bacteremia and the modified SIRS criteria were associated with lower age, an AP versus cUTI diagnosis, fluoroquinolone susceptibility, and the absence of a urinary tract anatomical disorder, each of which was univariably associated with higher percentages of successful response (data not shown). These incidental relationships reasonably explained the nonintuitive model results.

**Table 4.** Final Multivariable Logistic Regression Model for Successful Overall Response at TOC

Baseline Independent Variable	Parameter Estimate (Standard Error)	Odds Ratio (95% Confidence Interval)	Wald *P*-value
Intercept	0.594 (0.328)		
Urinary tract anatomical disorders	−0.734 (0.191)	0.480 (0.330–0.698)	< 0.001
Functional urinary tract or metabolic disorders	−0.629 (0.210)	0.533 (0.353–0.805)	0.003
CLcr > 50 mL/min	0.708 (0.272)	2.029 (1.190–3.459)	0.009
Fluoroquinolone susceptible phenotype	0.406 (0.184)	1.501 (1.047–2.153)	0.027
Bacteremia	0.571 (0.324)	1.770 (0.938–3.338)	0.08
Body mass index ≥ 32.2 kg/m^2^	−0.336 (0.232)	0.715 (0.454–1.126)	0.15

Abbreviations: CLcr, creatinine clearance; TOC, test-of-cure.

A total of only 3 of 70 potential interactions assessed across the multivariable models were found to be statistically significant at the 0.10 level. A statistically significant interaction with treatment group was found for instrumentation at baseline in models for clinical response at TOC and LFU, which directionally suggested reduced percentages of successful response only for ertapenem-treated patients. Trimethoprim-sulfamethoxazole resistance phenotype yielded a significant interaction via differences in directionality by treatment group in the model for clinical response at LFU. However, associations within each treatment group were not of sufficient magnitude to yield statistical significance. No other statistically significant interactions with treatment group were identified. Given the large number of potential interactions considered, the total number of 6 interactions identified as statistically significant was consistent with the expectation by random chance alone.

### Assessment of PK-PD Relationships in the Context of Multivariable Models

The final multivariable models for the efficacy endpoints were used to assess if PK-PD indices within treatment groups were associated with efficacy endpoints in the context of covariate adjustments for independent variables in the models and for any interacting variables identified.

Table 5 shows univariable and multivariable odds ratios for associations between efficacy endpoints and log-transformed continuous forms of tebipenem baseline MIC, free-drug plasma AUC, and AUC:MIC ratio × 1/τ for TBP-PI-HBr–treated patients. Similarly, Table 6 shows such statistics for associations between efficacy endpoints and log-transformed continuous forms of ertapenem baseline MIC and free-drug plasma %T > MIC for ertapenem-treated patients. Univariable and patient factor-adjusted associations between PK-PD indices for each agent and the efficacy endpoints were measured by odds ratios for dichotomous endpoints. In the multivariable context, neither PK-PD indices nor baseline MIC value for each agent in their continuous forms were predictive of efficacy at a 0.05 significance level. Results of the sensitivity analyses of the univariable and patient factor-adjusted relationships between the efficacy endpoints and ertapenem free-drug plasma %T > MIC demonstrated that, across 200 simulations of individual variability in PK parameters, there was no substantial deviation from the odds ratios reported in Table 6, with a maximum relative deviation of only 12.3%. Supplementary Table 10 and Supplementary Table 11 show similar such findings for analyses based on the evaluation of optimized 2-group forms of PK-PD indices and baseline MIC for tebipenem and ertapenem, respectively. These results suggest a lack of meaningful relationships between efficacy endpoints and the PK-PD indices for either the TBP-PI-HBr or ertapenem treatment group.

**Table 5.** Univariable and Multivariable Odds Ratios for Associations Between Efficacy Endpoints and Tebipenem Baseline MIC and Day 1 Free-Drug Plasma AUC and AUC:MIC Ratio × 1/τ

Efficacy Endpoint	Univariable/Multivariable	Odds Ratio for Successful Response (95% CI)		
		Tebipenem MIC^a^	Tebipenem Free-Drug Plasma AUC^b^	Tebipenem Free-Drug Plasma AUC:MIC Ratio × 1/τ^c^
Clinical response at TOC	Univariable	0.944 (0.705–1.264)	5.128 (0.682–38.56)	1.612 (0.686–3.789)
	Multivariable	1.255 (0.891–1.767)	6.552 (0.760–56.50)	0.799 (0.306–2.084)
Clinical response at LFU	Univariable	0.934 (0.722–1.209)	2.797 (0.476–16.43)	1.475 (0.681–3.194)
	Multivariable	1.060 (0.807–1.392)	2.908 (0.442–19.13)	1.033 (0.452–2.362)
Microbiologic response at TOC	Univariable	**0.794 (0.679–0.927)**	0.628 (0.236–1.666)	**1.719 (1.067–2.767)**
	Multivariable	0.866 (0.726–1.032)	0.565 (0.202–1.584)	1.277 (0.754–2.162)
Microbiologic response at LFU	Univariable	**0.805 (0.687–0.943)**	0.712 (0.264–1.921)	**1.697 (1.047–2.750)**
	Multivariable	0.879 (0.733–1.054)	0.780 (0.268–2.266)	1.338 (0.770–2.324)
Overall response at TOC	Univariable	**0.808 (0.692–0.943)**	0.699 (0.266–1.841)	**1.672 (1.042–2.683)**
	Multivariable	0.881 (0.739–1.050)	0.630 (0.225–1.761)	1.250 (0.738–2.116)

**Bolded** = significant at 0.05 level.

Abbreviations: AUC, area under the curve; CI, confidence interval; LFU, late follow-up; MIC, minimum inhibitory concentration; TOC, test-of-cure.

^a^Odds ratio for tebipenem MIC corresponds to a 1-dilution increase.

^b^Odds ratio for tebipenem free-drug plasma AUC corresponds to a 10-fold increase.

^c^Odds ratio for tebipenem free-drug plasma AUC:MIC ratio × 1/τ corresponds to a 10-fold increase.

**Table 6.** Univariable and Multivariable Odds Ratios for Associations Between Efficacy Endpoints and Ertapenem Baseline MIC and Day 1 Free-Drug Plasma %T > MIC

Efficacy Endpoint	Univariable/Multivariable	Odds Ratio for Successful Response (95% CI)	
		Ertapenem MIC^a^	Ertapenem Free-Drug Plasma %T > MIC^b^
Clinical response at TOC	Univariable	0.853 (0.699–1.041)	1.154 (0.896–1.486)
	Multivariable	0.747 (0.559–1.000)	1.221 (0.866–1.722)
Clinical response at LFU	Univariable	0.887 (0.763–1.030)	1.118 (0.921–1.357)
	Multivariable	0.866 (0.701–1.069)	1.161 (0.912–1.479)
Microbiologic response at TOC	Univariable	**0.847 (0.769–0.932)**	**1.191 (1.048–1.354)**
	Multivariable	0.919 (0.816–1.035)	1.121 (0.975–1.289)
Microbiologic response at LFU	Univariable	**0.871 (0.792–0.958)**	**1.162 (1.022–1.321)**
	Multivariable	0.902 (0.782–1.041)	1.131 (0.972–1.316)
Overall response at TOC	Univariable	**0.835 (0.759–0.919)**	**1.208 (1.062–1.374)**
	Multivariable	0.902 (0.800–1.015)	1.129 (0.981–1.299)

**Bolded** = significant at 0.05 level.

Abbreviations: CI, confidence interval; LFU, late follow-up; MIC, minimum inhibitory concentration; TOC, test-of-cure.

^a^Odds ratio for ertapenem MIC corresponds to a 1 dilution increase.

^b^Odds ratio for ertapenem free-drug plasma %T > MIC corresponds to a 10% increase.

## DISCUSSION

The analyses described herein were undertaken to achieve 3 objectives using pooled data from TBP-PI-HBr– or ertapenem-treated patients with cUTI/AP in the ADAPT-PO study. First, multivariable models were used to identify variables predictive of success or failure for various efficacy endpoints. Second, interactions between treatment groups and other variables were examined to understand if variables demonstrated differences in the magnitudes of their impacts on efficacy by treatment in the context of the multivariable models for efficacy. Third, relationships between PK-PD indices for tebipenem and ertapenem and efficacy endpoints within each treatment group were examined, also within the framework of multivariable models for efficacy.

The results based on the pooled data from both study arms demonstrated that certain patient factors were associated with lower probabilities of a successful response. Across all analyses using multivariable logistic regression models for the dichotomous efficacy endpoints, the presence of urinary tract anatomical disorders and/or functional urinary tract or metabolic disorders at baseline predicted reduced probabilities of success. These factors had a greater impact on the predicted probability of success for overall (composite) response at TOC and microbiologic response at TOC or LFU as compared to clinical response by more than 20%. Factors such as baseline CLcr > 50 mL/min and baseline pathogen susceptibility to fluoroquinolones were independently associated with higher probabilities of success for overall response at TOC and microbiologic response at TOC or LFU. However, having at least 1 Enterobacterales pathogen with the extended-spectrum beta-lactamase–positive phenotype predicted lower probabilities of success for microbiologic response at LFU and clinical response at TOC.

In most cases, interactions between patient variables and treatment group were not statistically significant when evaluated within the multivariable models. The few interactions that did emerge as statistically significant were in line with what would be expected by random chance alone, and none of these were expected a priori. Overall, the analysis of interactions between treatment group and other independent variables within the multivariable models did not yield meaningful insights. These findings suggested that patient factors influencing efficacy were similar across treatment groups.

No meaningful relationships between tebipenem and ertapenem free-drug plasma exposures and efficacy were identified. Tebipenem free-drug plasma AUC, AUC:MIC ratio × 1/τ, ertapenem free-drug plasma %T > MIC, and baseline MIC value for each drug did not show any association with efficacy endpoints when evaluated in the context of multivariable models. The likely reason for the lack of statistically significant clinical PK-PD relationships by treatment group is that the dosing regimens for TBP-PI-HBr and ertapenem were designed to result in PK-PD indices that were on the upper flat asymptote of such relationships. Therefore, these findings suggest that when dosing regimens are optimized based on nonclinical PK-PD targets for efficacy, patient factors are more predictive of response.

By definition, cUTIs occur in the presence of urinary tract anatomical or functional disorders, upper and lower genitourinary (GU) instrumentation such as stents and bladder catheterization, and/or metabolic disorders [8, 9, 27]. Previous prospective cUTI trials and observational data have shown that recurrent or persistent bacteriuria is frequently observed despite antibiotic therapy [1–14]. Additionally, recurrent or persistent bacteriuria is more common in patients with urinary tract anatomical disorders and/or functional urinary tract or metabolic disorders, or those with prior GU instrumentation [1, 8, 9, 28, 29].

The current analysis results add to the above-described findings by demonstrating similar associations of these risk factors with different efficacy endpoints in the setting of a large prospective clinical trial. We observed reduced success in patients with GU instrumentation compared to those without, but the univariable relationships were not statistically significant for most efficacy endpoints. Urinary tract anatomical disorders demonstrated greater magnitudes of association with efficacy endpoints than did instrumentation, although fewer patients had instrumentation compared to anatomical disorders, possibly influencing these findings.

Additionally, parallel analysis results showed no association between plasma antibiotic exposure during treatment and efficacy endpoints. This suggests that microbiologic (and to a lesser extent clinical) failure after the completion of treatment is more closely related to patient-specific anatomical and functional issues than with antibiotic efficacy, at least when dosing regimens providing optimized plasma antibiotic exposure are administered. This may be due to the urinary pathogens being sequestered in protected GU sites (eg, calculi, indwelling hardware, biofilms) during treatment, where slow bacterial replication occurs [8, 9, 30–36]. Another important factor could be the extent and duration of local antibiotic exposure in the urine [37–41].

One limitation of the analyses described herein is that the calculation of ertapenem free-drug plasma %T > MIC relied on dosing history, demographic information, and a population PK model, but in the absence of PK data from patients treated with ertapenem. However, since the multivariable models were not developed using ertapenem and tebipenem PK-PD, any conclusions regarding the associations between patient factors and study outcomes were not affected by limitations in calculating ertapenem exposures. Moreover, the results of sensitivity analyses demonstrated that variations in randomly assigned PK parameters did not affect the evaluation of model-adjusted relationships between ertapenem free-drug plasma %T > MIC and patient outcome.

In summary, the results of the multivariable analyses based on pooled data from TBP-PI-HBr– or ertapenem-treated patients with cUTI/AP in the ADAPT-PO study revealed associations between certain patient factors and reduced probabilities of successful response. Interactions between treatment group and other efficacy variables in the context of multivariable models were minimal. Assessments of relationships between PK-PD indices for tebipenem and ertapenem and efficacy endpoints within treatment groups suggested that drug exposure was not predictive of response, indicating that the study dosing regimens were optimized. Accordingly, reductions in overall and microbiologic response among patients with cUTI/AP were associated with anatomical or functional urinary tract disorders and other patient characteristics, rather than the extent or duration of plasma antibiotic exposure. These findings contribute to our understanding of factors that predict outcomes in patients with cUTI/AP and thus, have the potential to improve clinical trial design by informing clinical trial efficacy endpoints and/or inclusion/exclusion criteria.

## Supplementary Data

Supplementary materials are available at *Open Forum Infectious Diseases* online. Consisting of data provided by the authors to benefit the reader, the posted materials are not copyedited and are the sole responsibility of the authors, so questions or comments should be addressed to the corresponding author.

## Supplementary Material

ofae375_Supplementary_Data

## Notes

***Author Contributions.*** All authors were involved in the conceptualization and preparation of this manuscript.

***Financial support*.** Funding for this analysis was provided by Spero Therapeutics, Inc., Cambridge, MA.
