# Supplementary material for: Evaluation of Factors Predictive of Efficacy Among Patients With Complicated Urinary Tract Infection and/or Acute Pyelonephritis
Source: Open Forum Infect Dis. 2024 Jul 16;11(9):ofae375. doi: 10.1093/ofid/ofae375 (PMC11370788; doi:10.1093/ofid/ofae375)
Supplement: ofae375_Supplementary_Data [file ofae375_supplementary_data.pdf]

Supplementary Materials

Supplementary Methods

Ertapenem Population PK Model Description

The population PK model described by Lakota *et al*. was a linear three-compartment model with total body weight as a covariate on clearance and body surface area (BSA) as a covariate on central volume [1]. This model had been developed using data from subjects with normal renal function, which resulted in a lack of a significant correlation between renal function and ertapenem. Given that ertapenem clearance is known to be lower in patients with reduced renal function [2], the published model was refined in order to include a relationship between ertapenem clearance and creatinine clearance (CLcr) normalized to BSA (mL/min/1.73 m^2). This was accomplished by fitting a linear regression model to the observed clearance and CLcr values from Mistry *et al*. [2] (**Supplementary Figure 1**) and including the slope derived from that fit into the typical value equation for ertapenem clearance. The typical value equations for this refined population PK model are provided below:

$CL\ (L/h) = [1.71 + 0.01143 * (CLcr-110)] * (WTKG/95.9)^{0.278}$

$Vc\ (L) = 4.76 * (BSA/2.06)^{1.86}$

CLd1 (L/h) = 6.71

Vp1 (L) = 2.96

CLd2 (L/h) = 0.296

Vp2 (L) = 1.1

Where CL is the typical value of ertapenem clearance; CLcr is creatinine clearance in mL/min/1.73 m^2; WTKG is body weight in kg; Vc is volume of the central compartment; BSA is body surface area in m^2; Vp1 is volume of the first peripheral compartment; CLd1 is the clearance into Vp1; Vp2 is volume of the second peripheral compartment; and CLd2 is the clearance into Vp2. The interindividual variability estimates for ertapenem CL and Vc/Vp1 were 9.57% and 7.92%, respectively. Note that the relationship between ertapenem CL and CLcr was modified from the regression equation in **Supplementary Figure 1** to reflect the typical value of ertapenem CL in the subjects with normal renal function included in the dataset used to development the model from Lakota *et al.* [1].

Predictive Performance of the Population Pharmacokinetic Model for Subjects Without Ertapenem Concentration-Time Data

A validation exercise was performed to determine whether the ertapenem population PK model can be used to generate PK parameters and ertapenem exposures using dosing history and demographic information alone for patients in the absence of concentration-time data. The goal of this exercise was to confirm that using the typical value of the PK parameters (i.e., without invoking random interindividual variability) is a valid approach for estimating exposure in patients for whom drug concentration data were unavailable. This exercise was carried out using R version 4.0.4 [3].

For the first set of simulations, 687 ertapenem-treated patients with cUTI/AP from the ADAPT-PO study were replicated five times in order to generate a simulated population that contained 3,435 simulated patients. Individual PK parameters for ertapenem were calculated for each simulated patient using demographic values derived from the observed ADAPT-PO study population and the population PK model. First, typical PK values for each simulated patient were calculated using patient characteristics that were included as covariates in the population PK model. Individual PK parameter values for each simulated patient were then generated by applying an individual specific random effect (η) to each patient's typical PK value. Each simulated patient's η value was drawn from a log-normal distribution with a mean of zero and a variance derived from the population PK model for each parameter with associated IIV. Given that each patient in a given simulated population was assigned a set of PK parameters via Monte Carlo simulation, the individual patient replicates were subsequently considered to be distinct simulated patients. Ertapenem total-drug plasma concentration-time profiles from 0 to 24 hours were generated for each simulated patient based on the dosing history of the originally observed patients. Ertapenem free-drug plasma %T>MIC from 0 to 24 hours was determined for each simulated patient by counting the total number of free-drug concentrations that were above a given MIC value, multiplying this number by the time interval between simulated concentrations (0.1 hour), and then dividing this product by 24 hours. Ertapenem free-drug plasma %T>MIC was determined for fixed ertapenem MIC values based on the observed range of MIC values for the collections of Enterobacterales. These values were defined as the "true" free-drug plasma %T>MIC values (free-drug plasma %T>MIC$_{true}$).

For the second set of simulations, the same process was performed as the first set of simulations except that random effects were not implemented. Thus, the typical PK parameter value (e.g., population mean) was calculated for each simulated patient using only their specific demographic information in the population PK model. Ertapenem free-drug plasma %T>MIC were subsequently generated using the same process described for the first set of simulations. These values were defined as the "population mean" free-drug plasma %T>MIC values (free-drug plasma %T>MIC$_{population\ mean}$).

The free-drug plasma %T>MIC$_{population\ mean}$ values were then compared to the free-drug plasma %T>MIC$_{true}$ values for each simulated patient by calculating the percent predicted error (PE%) which is free-drug plasma %T>MIC$_{population\ mean}$ - free-drug plasma %T>MIC$_{true}$. Summary statistics of the percent predicted error (PE%) and absolute predicted error (|PE%|) were calculated and used to assess bias and precision of the %T>MIC$_{population\ mean}$ across MIC values ranging from 0.002 to 16 mg/L, respectively. As a general guideline, if the mean PE% was ± 5% and |PE%| was less than 10%, it was to be deemed that the population PK model for ertapenem can provide a reasonable estimation of free-drug plasma %T>MIC for the patients enrolled in the ADAPT-PO study who received ertapenem and had dosing history and demographic information available but did not have ertapenem PK data collected.

Supplementary Results

The use of the typical values of the PK parameters (i.e., the population mean values based on relevant patient characteristics) for ertapenem when estimating %T/MIC provides unbiased and reasonably precise estimates of free-drug plasma %T>MIC. The

bias and precision for the free-drug plasma %T>MIC by MIC value are provided in **Supplementary Table 12**. Ertapenem Free-Drug Plasma %T>MIC$_{\text{population mean}}$ is shown in **Supplementary Figure 2**. A box-plot of the distribution of PE% by MIC value (**Supplementary Figure 3**) showed that the bias was minimal (median PE% was 0%) across all MIC values. For MIC values ranging from 0.002 to 0.12 µg/mL, precision was high as indicated by the collapsed boxplots which is attributed to the concentration profile being entirely above the MIC threshold leading to free-drug plasma %T>MIC value of 100% for both estimated and true values. For MIC values ranging from 0.25 to 2 µg/mL, precision decreased as indicated by the wider boxplots but was still within the acceptable range of < 10% (median |PE%| was less than 4.15%). For MIC values ranging from 4 to 16 µg/mL, the precision increased as the median |PE%| was less than 1.25% which is attributed to the concentration profile being entirely below the MIC threshold leading to a free-drug plasma %T>MIC value of 0% for both estimated and true values. Therefore, the approach of using the typical values of the PK parameters from the model, as opposed to invoking randomly variability, was deemed acceptable for estimating ertapenem free-drug plasma %T>MIC values for use in the primary PK-PD analyses. The appropriateness of this approach is further justified based on the results of the sensitivity analyses described in the results section of the publication.

Supplementary Table 1. Summary statistics for categorical baseline patient demographic and disease characteristics based on data from patients in the ME analysis population infected with Enterobacterales pathogen(s) at baseline, by treatment group and pooled

Baseline characteristic	% (n/N) of patients by treatment		
	**TBP-PI-HBr (n=366)**	**Ertapenem (n=378)**	**Pooled (n=744)**
Age group			
≥ 18 to < 65 years	54.1 (198/366)	52.9 (200/378)	53.5 (398/744)
≥ 65 years	45.9 (168/366)	47.1 (178/378)	46.5 (346/744)
Sex			
Male	43.2 (158/366)	37.3 (141/378)	40.2 (299/744)
Female	56.8 (208/366)	62.7 (237/378)	59.8 (445/744)
CLcr > 50 mL/min[a]	87.2 (319/366)	91.5 (346/378)	89.4 (665/744)
Ethnicity			
Hispanic or Latino	1.6 (6/366)	0.5 (2/378)	1.1 (8/744)
Not Hispanic or Latino	98.4 (360/366)	99.5 (376/378)	98.9 (736/744)
Race			
Asian	0 (0/366)	0.3 (1/378)	0.1 (1/744)
African American or Black	0.8 (3/366)	0 (0/378)	0.4 (3/744)
White	99.2 (363/366)	99.7 (377/378)	99.5 (740/744)
Region			
Central and Eastern Europe	98.4 (360/366)	98.9 (374/378)	98.7 (734/744)
South Africa	0.8 (3/366)	0.3 (1/378)	0.5 (4/744)
USA	0.8 (3/366)	0.8 (3/378)	0.8 (6/744)
Diagnosis			
cUTI	47.5 (174/366)	50.0 (189/378)	48.8 (363/744)
AP	52.5 (192/366)	50.0 (189/378)	51.2 (381/744)
Specimen type[b]			
Blood	10.1 (37/366)	12.2 (46/378)	11.2 (83/744)
Urine	89.9 (329/366)	87.8 (332/378)	88.8 (661/744)

Supplementary Table 1. Summary statistics for categorical baseline patient demographic and disease characteristics based on data from patients in the ME analysis population infected with Enterobacterales pathogen(s) at baseline, by treatment group and pooled

Baseline characteristic	% (n/N) of patients by treatment		
	**TBP-PI-HBr (n=366)**	**Ertapenem (n=378)**	**Pooled (n=744)**
ESBL resistance phenotype[c]			
Negative	72.7 (266/366)	77.5 (293/378)	75.1 (559/744)
Positive	27.3 (100/366)	22.5 (85/378)	24.9 (185/744)
Fluoroquinolone resistance phenotype[d]			
Susceptible	60.1 (220/366)	61.9 (234/378)	61.0 (454/744)
Non-susceptible	39.9 (146/366)	38.1 (144/378)	39.0 (290/744)
TMP-SMX resistance phenotype[e]			
Susceptible	43.2 (158/366)	43.7 (165/378)	43.4 (323/744)
Resistant	56.8 (208/366)	56.3 (213/378)	56.6 (421/744)
Bacteremia	10.7 (39/366)	12.4 (47/378)	11.6 (86/744)
Urinary tract anatomical disorders	53.0 (194/366)	53.2 (201/378)	53.1 (395/744)
Functional urinary tract or metabolic disorders	21.6 (79/366)	19.8 (75/378)	20.7 (154/744)
Patients with a history of diabetes	20.5 (75/366)	17.5 (66/378)	19.0 (141/744)
Urinary tract instrumentation/procedures	16.4 (60/366)	9.8 (37/378)	13.0 (97/744)
Modified SIRS criteria at baseline	24.0 (88/366)	18.3 (69/378)	21.1 (157/744)
Prior systemic antibiotics	3.8 (14/366)	4.8 (18/378)	4.3 (32/744)
Risk factors[f]			
At least one risk factor	86.6 (317/366)	86.2 (326/378)	86.4 (643/744)
At least two risk factors	63.4 (232/366)	61.9 (234/378)	62.6 (466/744)

a. Creatinine clearance (CLcr) was calculated by Cockcroft Gault equation using serum creatinine data collected at baseline from the local laboratory [4].
b. Based on the selected analysis pathogen.
c. ESBL resistance phenotype was defined as positive if ceftazidime MIC ≥ 2 µg/mL (or ceftriaxone MIC ≥ 2 µg/mL if ceftazidime susceptibility was not available).
d. Fluoroquinolone resistance phenotype was defined as non-susceptible if levofloxacin MIC ≥ 1 µg/mL.
e. TMP-SMX resistance phenotype was defined as non-susceptible if TMP-SMX MIC ≥ 4 µg/mL.
f. Baseline risk factors include the following: age ≥ 65 years, cUTI, bacteremia at baseline, modified SIRS criteria at baseline, urinary tract anatomical disorders, functional urinary tract or metabolic disorders, or urinary tract instrumentation/procedure at baseline.

Supplementary Table 2. Summary statistics for continuous baseline patient demographic and disease characteristics based on data from patients in the ME analysis population infected with Enterobacterales pathogen(s) at baseline, by treatment group and pooled

Baseline characteristic	TBP-PI-HBr (n=366)		Ertapenem (n=378)		Pooled (n=744)	
	Mean (%CV)	Median (Min, Max)	Mean (%CV)	Median (Min, Max)	Mean (%CV)	Median (Min, Max)
Age (years)	58.0 (32.2)	63 (18, 91)	58.6 (30.7)	63 (18, 91)	58.3 (31.4)	63 (18, 91)
Total body weight (kg)	77.6 (22.3)	75.1 (45, 142)	77.1 (19.9)	76 (43, 130.6)	77.3 (21.1)	75.5 (43, 142)
Body surface area (m^2)	1.87 (12.0)	1.85 (1.32, 2.70)	1.86 (10.5)	1.85 (1.40, 2.51)	1.86 (11.2)	1.85 (1.32, 2.70)
Body mass index (kg/m^2)	27.4 (20.5)	26.4 (16.0, 57.9)	27.4 (18.6)	27.1 (15.8, 46.1)	27.4 (19.5)	26.9 (15.8, 57.9)
CLcr (mL/min)[a]	89.8 (43.6)	84 (32, 304.9)	87.0 (38.0)	83 (30.3, 273)	88.4 (40.9)	83.75 (30.3, 304.9)

a. Creatinine clearance (CLcr) was calculated by Cockcroft Gault equation using serum creatinine data collected at baseline from the local laboratory [4].

Supplementary Table 3. Listing of terms used to define urinary tract anatomical disorders, functional urinary tract or metabolic disorders, and urinary tract instrumentation/procedures

Variable	Terms satisfying the definition	
Urinary tract anatomical disorders	Benign neoplasm of bladder	Neurogenic bladder
	Benign prostatic hyperplasia	Pelvi-ureteric obstruction
	Benign renal neoplasm	Prostate cancer
	Bladder cancer	Prostate cancer metastatic
	Bladder diverticulum	Prostatic adenoma
	Bladder dysfunction	Pyelocaliectasis
	Bladder leukoplakia	Renal atrophy
	Bladder neck sclerosis	Renal cancer
	Bladder neoplasm	Renal cancer stage I
	Bladder obstruction	Renal cyst
	Bladder stenosis	Renal fusion anomaly
	Bladder transitional cell carcinoma	Renal hamartoma
	Calculus bladder	Renal hypertrophy
	Calculus urinary	Renal hypoplasia
	Chronic kidney disease	Renal neoplasm
	Congenital megaureter	Single functional kidney
	Diabetic nephropathy	Stress urinary incontinence
	Hydronephrosis	Tubulointerstitial nephritis
	Hypertonic bladder	Ureteral cyst
	Hypotonic urinary bladder	Ureteric stenosis
	Ischaemic nephropathy	Ureterocele
	Kidney duplex	Ureterolithiasis
	Kidney fibrosis	Urethral meatus stenosis
	Kidney malrotation	Urethral perforation
	Kidney small	Urethral stenosis
	Nephrolithiasis	Urinary incontinence
	Nephroptosis	Urinary retention
	Nephrosclerosis	Urinary tract malformation

Supplementary Table 3. Listing of terms used to define urinary tract anatomical disorders, functional urinary tract or metabolic disorders, and urinary tract instrumentation/procedures

Variable	Terms satisfying the definition	
Functional urinary tract or metabolic disorders	Carbohydrate metabolism disorder	Hyperglycaemia
	Cauda equina syndrome	Insulin resistance
	Chronic inflammatory demyelinating polyradiculoneuropathy	Insulin-requiring type 2 diabetes mellitus
	Diabetes mellitus	Paraparesis
	Diabetic encephalopathy	Paraplegia
	Diabetic neuropathy	Polyneuropathy
	Glucose tolerance impaired	Polyneuropathy idiopathic progressive
	Hemiparesis	Type 1 diabetes mellitus
		Type 2 diabetes mellitus
Urinary tract instrumentation/procedures	Benique dilation of the bladder neck sclerosis	Meatotomy
	Benique dilation of the urethral strictures	Nephrostomy
	Bladder catheter removal	Nephrostomy tube
	Bladder catheter replacement	Nephrostomy tube removal
	Bladder catheterization	Obturatio cystostome
	Bladder neck dilations with benique dilations	Percutaneous nephrolithotripsy
	Condom catheter	Percutaneous nephrolithotripsy (left and right)
	Contact laser lithotripsy of concretion of the right ureter	Percutaneous nephrolithotripsy (left)
	Cystostomy	Percutaneous nephrolithotripsy (right)
	Dilatatio uretherae	Reanastomosis urhetrae
	Dilatatio uretra	Right extracorporeal shock wave lithotripsy
	Dilatation of the bladder neck stricture	Stent
	Endoscopic cystolytotripsy	Suprapubic bladder catheter
	Endoscopic small bladder stones extraction	Transurethral balloon dilation of the vesicourethral anastomotic stricture
	ESWL[a]	Transurethral bladder resection
	ESWL[a] of left kidney	Transurethral resection of bladder leukoplakia
	ESWL[a] of right kidney	Transurethral resection of the prostate
	ESWL[a] of the left side	Ureteral catheter
	Extracorporeal lithotripsy (ESWL)	Ureteral catheterization
	Extracorporeal shock wave lithotripsy	Ureteral stent insertion
	Flushing of the bladder	Ureteral stent removal
	Instrumental dilatation of the urethra with urethral bougie	Ureteric calculus removal
	Left sided nephrectomy	Ureterolithotripsy
	Left ureter bougienage	

Supplementary Table 3. Listing of terms used to define urinary tract anatomical disorders, functional urinary tract or metabolic disorders, and urinary tract instrumentation/procedures

Variable	Terms satisfying the definition	
Urinary tract Instrumentation/procedures	Lithoclast endolithotripsio	Ureterorenoscopy with kidney stone extraction
	Lithotripsy	Ureterostoma tube
	Urethral bladder catheter	Urethral dilations with benique dilatators
	Urethral bougienage	Urethral stricture dilations with benique dilatators
	Urethral dilation	Urethrectomy
	Ureterorenoscopic lithotripsy	Urethrocystoscopy
	Ureterorenoscopy with kidney stone extraction	Urodynamic

a. ESWL represents extracorporeal shock wave lithotripsy.

Supplementary Table 4. Assessments of univariable associations between efficacy endpoints and candidate independent variables for the multivariable analyses

Univariable relationships and interactions with treatment identified as significant at individual levels of 0.05 and 0.10, respectively					
**Independent variable**	**Clinical response**		**Microbiologic response**		**Overall response**
	**TOC**	**LFU**	**TOC**	**LFU**	**TOC**
Age		T, P	T, P	T, P	T, P
Sex		E, P	T, P	T, P	T, P
Body mass index			E, P	E, P	E, P
CLcr > 50 mL/min	T	T		T, P	T, P
Ethnicity					
Race					
Region					
Diagnosis	T, P	T, P	T, E, P	T, E, P	T, E, P
ESBL resistance phenotype	T		T, P	T, E, P	T, P
Fluoroquinolone resistance phenotype	P		T, E, P	T, E, P	T, E, P
TMP-SMX resistance phenotype					
Bacteremia			**P**		**T, P**
Urinary tract anatomical disorders	T, P	T, E, P	T, E, P	T, E, P	T, E, P
Functional urinary tract or metabolic disorders	T, P	T, P	T, E, P	T, E, P	T, E, P
Urinary tract instrumentation/procedures		E		P	
Modified SIRS criteria		**E, P**	**E, P**	**T, P**	**E, P**
Prior systemic antibiotics					
At least one risk factor		P	P	P	E, P
At least two risk factors		T, P	T, E, P	T, E, P	T, E, P

Note: A gray-shaded cell indicates that an interaction with treatment group was significant at the 0.10 level. Bolded letters indicated the groups for which non-intuitive findings were found. T = TBP-PI-HBr. E = Ertapenem. P = Pooled. TOC=Test-of-Cure assessed on Day 19 ± 2; LFU= Late Follow-Up assessed on Day 25 ± 2.

Supplementary Table 5. Final multivariable logistic regression model for successful clinical response at TOC

Baseline independent variable	Parameter estimate (SE)	Odds ratio (95% CI)	Wald P-value
Intercept	4.018 (0.403)		
Urinary tract anatomical disorders	-0.925 (0.449)	0.397 (0.165 – 0.955)	0.039
Functional urinary tract or metabolic disorders	-0.617 (0.396)	0.540 (0.248 – 1.174)	0.12
ESBL-positive phenotype	-0.587 (0.384)	0.556 (0.262 – 1.180)	0.13

Supplementary Table 6. Final multivariable logistic regression model for successful clinical response at LFU

**Baseline independent variable**	**Parameter estimate (SE)**	**Odds ratio (95% CI)**	**Wald P-value**
Intercept	3.420 (0.379)		
Urinary tract anatomical disorders	-1.388 (0.405)	0.250 (0.113 – 0.552)	0.001
Modified SIRS criteria at baseline	0.957 (0.619)	2.605 (0.775 – 8.754)	0.12
Prior systemic antibiotics	-1.014 (0.588)	0.363 (0.115 – 1.149)	0.08

Supplementary Table 7. Final multivariable logistic regression model for successful microbiologic response at TOC

**Baseline independent variable**	**Parameter estimate (SE)**	**Odds ratio (95% CI)**	**Wald P-value**
Intercept	0.696 (0.331)		
Urinary tract anatomical disorders	-0.741 (0.194)	0.477 (0.326 – 0.696)	<0.001
Functional urinary tract or metabolic disorders	-0.703 (0.207)	0.495 (0.330 – 0.743)	0.001
Fluoroquinolone susceptibility	0.431 (0.185)	1.539 (1.071 – 2.212)	0.020
CLcr > 50 mL/min	0.622 (0.273)	1.862 (1.090 – 3.182)	0.023
Bacteremia	0.476 (0.323)	1.609 (0.854 – 3.032)	0.14

Supplementary Table 8. Final multivariable logistic regression model for successful microbiologic response at LFU

**Baseline independent variable**	**Parameter estimate (SE)**	**Odds ratio (95% CI)**	**Wald P-value**
Intercept	0.744 (0.309)		
Urinary tract anatomical disorders	-0.784 (0.190)	0.457 (0.315 – 0.662)	<0.001
CLcr > 50 mL/min	0.996 (0.280)	2.709 (1.565 – 4.687)	<0.001
Functional urinary tract or metabolic disorders	-0.681 (0.213)	0.506 (0.333 – 0.768)	0.001
ESBL-positive phenotype	-0.409 (0.200)	0.664 (0.449 – 0.984)	0.041
Body mass index ≥ 26.0 kg/m^2	-0.317 (0.189)	0.729 (0.503 – 1.056)	0.09

Supplementary Table 9. Selected associations with bacteremia and modified SIRS criteria

Baseline characteristic	% (n/N) of patients by criteria			
	**Without bacteremia at baseline (n=658)**	**With bacteremia at baseline (n=86)**	**SIRS criteria not met at baseline (n=587)**	**SIRS criteria met at baseline (n=157)**
Age group				
≥ 18 to < 65 years	51.8 (341/658)	66.3 (57/86)	47.9 (281/587)	74.5 (117/157)
≥ 65 years	48.2 (317/658)	33.7 (29/86)	52.1 (306/587)	25.5 (40/157)
Diagnosis at baseline				
cUTI	51.1 (336/658)	31.4 (27/86)	56.0 (329/587)	21.7 (34/157)
AP	48.9 (322/658)	68.6 (59/86)	44.0 (258/587)	78.3 (123/157)
Fluoroquinolone resistance phenotype[a]				
Susceptible	58.7 (386/658)	79.1 (68/86)	56.9 (334/587)	76.4 (120/157)
Non-susceptible	41.3 (272/658)	20.9 (18/86)	43.1 (253/587)	23.6 (37/157)
Urinary tract anatomical disorders	55.3 (364/658)	36.0 (31/86)	60.1 (353/587)	26.8 (42/157)

a. Fluoroquinolone resistance phenotype was defined as non-susceptible if levofloxacin MIC ≥ 1 μg/mL.

Supplementary Table 10. Univariable and multivariable odds ratios for associations between efficacy endpoints and optimized two-group forms of tebipenem baseline MIC and Day 1 free-drug plasma AUC and AUC:MIC ratio•1/τ

Efficacy Endpoint	Variable	Optimized threshold	% (n/N) below the threshold	% (n/N) at or above the threshold	Univariable odds ratio for successful response (95% CI)	Covariate-adjusted odds ratio for successful response (95% CI)
Clinical response at TOC	Tebipenem MIC (µg/mL)	0.03	93.5 (202/216)	94.4 (117/124)	1.158 (0.455–2.952)	2.526 (0.863 – 7.395)
	Tebipenem AUC (mg•h/L)	49.2	92.3 (227/246)	97.9 (92/94)	3.850 (0.879 – 16.86)	4.951 (1.074 – 22.83)
	Tebipenem AUC:MIC ratio•1/τ	300.2	91.7 (187/204)	97.1 (132/136)	3.000 (0.987 – 9.199)	2.231 (0.685 – 7.270)
Clinical response at LFU	Tebipenem MIC (µg/mL)	0.03	91.2 (186/204)	92.2 (107/116)	1.151 (0.499 – 2.651)	1.569 (0.649 – 3.794)
	Tebipenem AUC (mg•h/L)	30.8	88.4 (107/121)	93.5 (186/199)	1.872 (0.848 – 4.131)	1.928 (0.846 – 4.394)
	Tebipenem AUC:MIC ratio•1/τ	372.8	89.3 (201/225)	96.8 (92/95)	3.662 (1.075 – 12.47)	3.160 (0.891 – 11.21)
Microbiologic response at TOC	Tebipenem MIC (µg/mL)	0.03	72.7 (157/216)	59.7 (74/124)	0.556 (0.349 – 0.887)	0.709 (0.422 – 1.192)
	Tebipenem AUC (mg•h/L)	25.1	76.0 (57/75)	65.7 (174/265)	0.604 (0.336 – 1.087)	0.595 (0.323 – 1.097)
	Tebipenem AUC:MIC ratio•1/τ	118.7	59.1 (52/88)	71.0 (179/252)	1.698 (1.025 – 2.812)	1.310 (0.754 – 2.279)
Microbiologic response at LFU	Tebipenem MIC (µg/mL)	0.03	70.1 (143/204)	57.8 (67/116)	0.583 (0.363 – 0.938)	0.752 (0.439 – 1.288)
	Tebipenem AUC (mg•h/L)	42.6	68.5 (139/203)	60.7 (71/117)	0.711 (0.442 – 1.142)	0.823 (0.495 – 1.368)
	Tebipenem AUC:MIC ratio•1/τ	445.8	62.9 (156/248)	75.0 (54/72)	1.769 (0.978 – 3.199)	1.606 (0.838 – 3.078)
Overall response at TOC	Tebipenem MIC (µg/mL)	0.03	70.8 (153/216)	59.7 (74/124)	0.609 (0.383 – 0.969)	0.782 (0.465 – 1.315)
	Tebipenem AUC (mg•h/L)	25.1	75.7 (56/74)	64.3 (171/266)	0.579 (0.322 – 1.041)	0.587 (0.318 – 1.083)
	Tebipenem AUC:MIC ratio•1/τ	445.8	63.9 (168/263)	76.6 (59/77)	1.854 (1.033 – 3.326)	1.564 (0.842 – 2.905)

Note: Shading indicates statistical significance at an individual 0.05 level.

Supplementary Table 11. Univariable and multivariable odds ratios for associations between efficacy endpoints and optimized two-group forms of ertapenem baseline MIC and Day 1 free-drug plasma %T>MIC

Efficacy Endpoint	Variable	Optimized threshold	% (n/N) below the threshold	% (n/N) at or above the threshold	Univariable odds ratio for successful response (95% CI)	Covariate-adjusted odds ratio for successful response (95% CI)
Clinical response at TOC	Ertapenem MIC (µg/mL)	0.015	98.6 (208/211)	94.8 (127/134)	0.262 (0.066 – 1.030)	0.215 (0.046 – 1.000)
	Ertapenem %T>MIC	99.6	93.8 (30/32)	97.4 (305/313)	2.542 (0.516 – 12.52)	3.356 (0.357 – 31.52)
Clinical response at LFU	Ertapenem MIC (µg/mL)	0.03	94.6 (229/242)	90.0 (72/80)	0.511 (0.204 – 1.282)	0.556 (0.188 – 1.648)
	Ertapenem %T>MIC	99.6	90.0 (27/30)	93.8 274/292)	1.691 (0.468 – 6.111)	1.781 (0.356 – 8.921)
Microbiologic response at TOC	Ertapenem MIC (µg/mL)	0.015	81.5 (172/211)	66.4 (89/134)	0.448 (0.272 – 0.739)	0.652 (0.350 – 1.217)
	Ertapenem %T>MIC	99.6	50.0 (16/32)	78.3 (245/313)	3.603 (1.714 – 7.576)	2.129 (0.929 – 4.879)
Microbiologic response at LFU	Ertapenem MIC (µg/mL)	0.015	73.7 (146/198)	60.5 (75/124)	0.545 (0.338 – 0.880)	0.702 (0.367 – 1.345)
	Ertapenem %T>MIC	99.6	43.3 (13/30)	71.2 (208/292)	3.238 (1.506 – 6.961)	2.454 (0.942 – 6.392)
Overall response at TOC	Ertapenem MIC (µg/mL)	0.015	81.0 (171/211)	63.4 (85/134)	0.406 (0.248 – 0.664)	0.563 (0.305 – 1.039)
	Ertapenem %T>MIC	99.6	46.9 (15/32)	77.0 (241/313)	3.794 (1.805 – 7.971)	2.227 (0.968 – 5.119)

Note: Shading indicates statistical significance at an individual 0.05 level.

Supplementary Table 12. Median PE% and |PE%| for estimates of ertapenem free-drug plasma %T>MIC for simulated patients by MIC value

MIC (mg/L)	Bias (PE%[a])	Precision (|PE%|)
0.002	0	0
0.004	0	0
0.008	0	0
0.015	0	0
0.03	0	0
0.06	0	0
0.12	0	0
0.25	0	2.49
0.5	0	4.15
1	0	3.32
2	0	2.08
4	0	1.25
8	0	0.829
16	0	0

a. PE% = ertapenem free-drug plasma $\%T>MIC_{population\ mean}$ -free-drug plasma $\%T>MIC_{true}$.

Supplementary Figure 1. Relationship between ertapenem clearance and creatinine clearance using data from Mistry *et al*. [2]

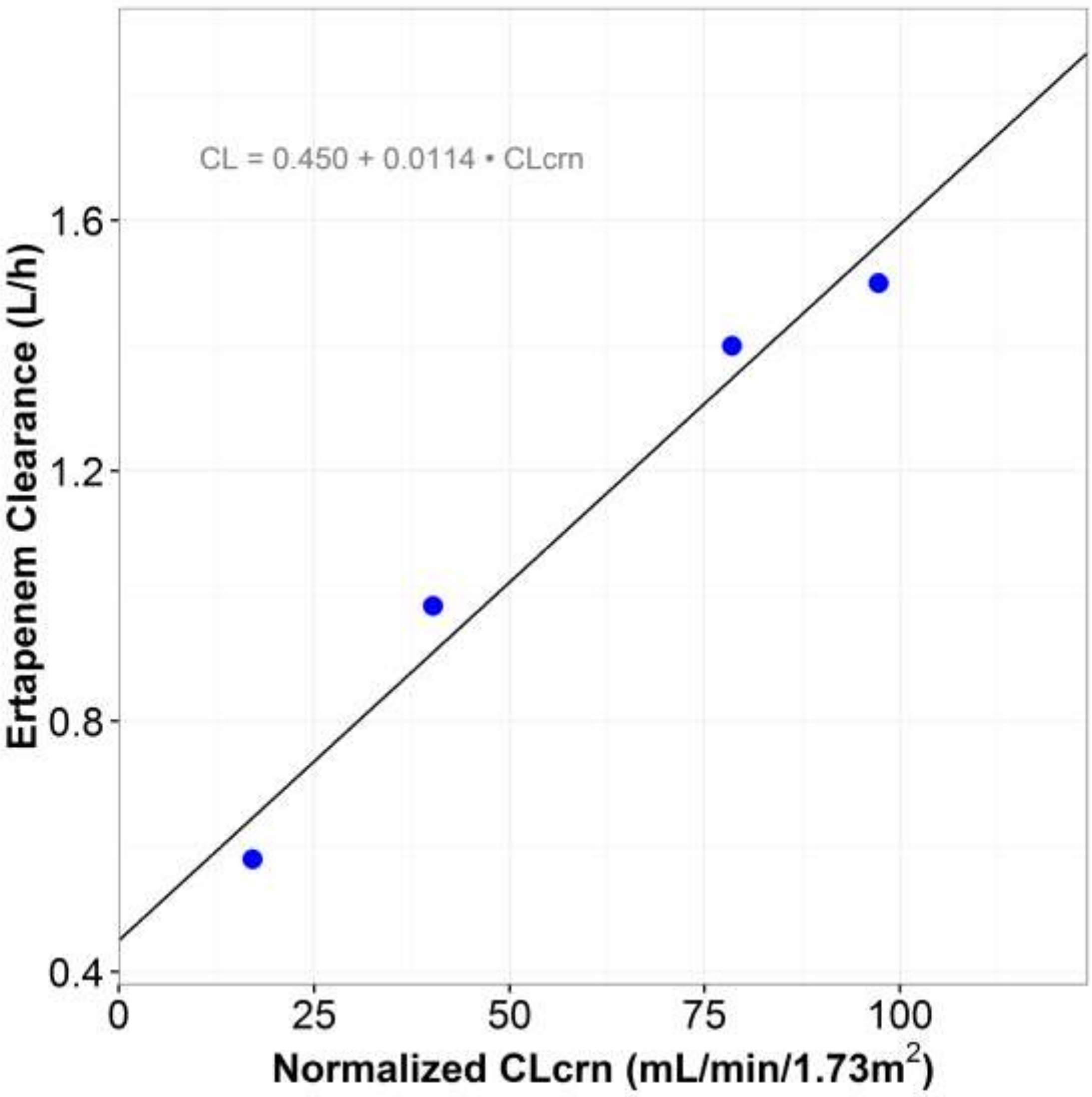

Supplementary Figure 2. Ertapenem Free-Drug Plasma %T>MIC$_{population\ mean}$

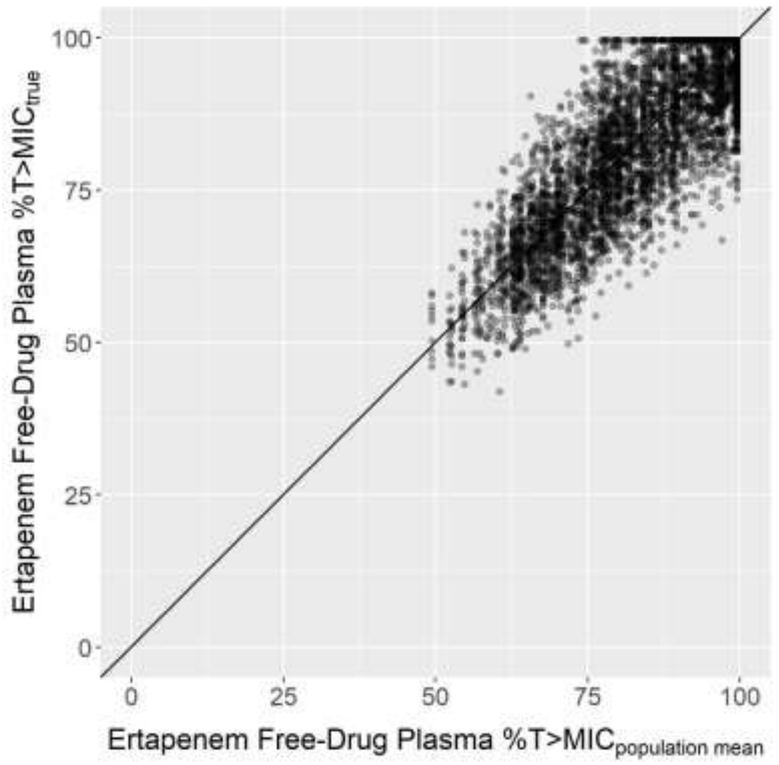

Note: Line represents the line of identity.

Supplementary Figure 3. Box plot of the PE% for ertapenem free-drug plasma %T>MIC by MIC value

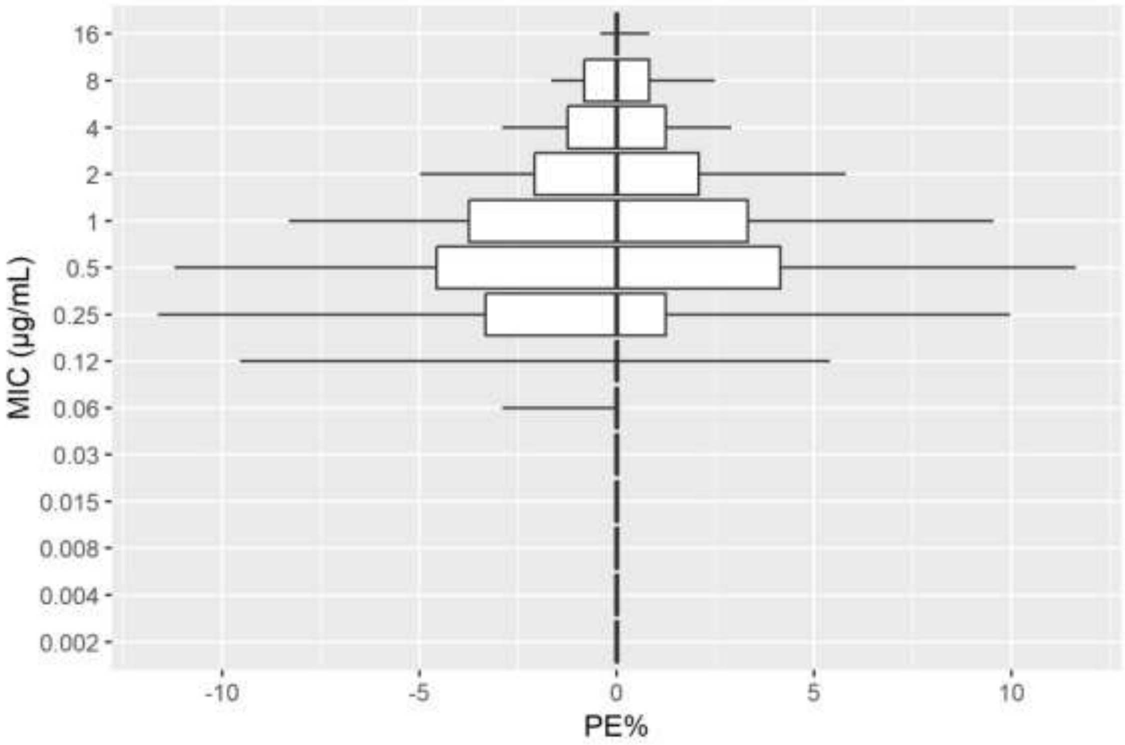

Note: Center line represents the median value, the box boundaries represent the 25th and 75th quantiles and the whiskers represent the 5th and 95th quantiles. PE% = ertapenem free-drug plasma $\%T>MIC_{\text{population mean}}$ – free-drug plasma $\%T>MIC_{\text{true}}$.

References

1. Lakota EA, Landersdorfer CB, Zhang L, *et al.* Population pharmacokinetic analyses for ertapenem in subjects with a wide range of body sizes. Antimicrob Agents Chemother. 2018;62(10):e00784-18.
2. Mistry GC, Majumdar AK, Swan S, *et al*. Pharmacokinetics of ertapenem in patients with varying degrees of renal insufficiency and in patients on hemodialysis. J Clin Pharmacol. 2006;46(10):1128-1138.
3. R [computer program]. Version 4.0.4. Vienna, Austria: R Development Core Team; R Foundation for Statistical Computing; 2021.
4. Cockcroft DW, Gault MH. Prediction of creatinine clearance from serum creatinine. Nephron. 1976;16:31-41.
